# Cellular senescence in brain aging and neurodegeneration: from molecular mechanisms to translational opportunities

**DOI:** 10.3389/fncel.2026.1805691

**Published:** 2026-05-19

**Authors:** Yahveth Cantero-Fortiz, Christopher Butler, Xavier Montalbán, Mercè Boada

**Affiliations:** 1 Ace Alzheimer Center Barcelona, Universitat Internacional de Catalunya, Barcelona, Spain; 2 Networking Research Center on Neurodegenerative Diseases (CIBERNED), Instituto de Salud Carlos III, Madrid, Spain; 3 The George Institute for Global Health, Imperial College London, London, United Kingdom; 4 Department of Brain Sciences, Imperial College London, London, United Kingdom; 5 Vall d'Hebron Hospital Universitari, Barcelona, Spain

**Keywords:** Alzheimer’s disease, brain aging, cellular senescence, Parkison’s disease, SASP, neurodegeneration

## Abstract

Aging remains the predominant risk factor for Alzheimer’s disease (AD) and other neurodegenerative disorders, yet the mechanisms linking systemic aging to brain dysfunction remain incompletely understood. Cellular senescence, a state of stable cell-cycle arrest coupled with metabolic and secretory reprogramming, has emerged as a pivotal and context-dependent driver of brain aging. Accumulation of senescent glial cells (astrocytes, microglia, and oligodendrocyte progenitors) and emerging evidence of “neurescence” in post-mitotic neurons contribute to neuroinflammation, impaired proteostasis, and synaptic dysfunction. This review synthesizes molecular, cellular, and translational findings that reframe senescence as an active process shaping brain vulnerability. We discuss SASP-mediated neurotoxicity, crosstalk among senescent glial subtypes, and context-specific pathways (NF-κB, p38 MAPK, mTOR, cGAS–STING) as therapeutic targets. Senomorphic and senolytic strategies, alongside emerging systemic interventions such as therapeutic plasma exchange with albumin replacement, are evaluated for their potential to mitigate senescence burden and restore homeostasis. Integrating evidence from fluid, imaging, and multi-omic biomarkers, we highlight how senescence can now be monitored *in vivo* and stratified across disease stages. Multi-omic and spatial transcriptomic data reveal that central and peripheral senescence signatures only partially overlap, suggesting bidirectional communication across the brain–body axis. This systemic dimension raises key questions about whether modifying peripheral senescence or proteostasis could reshape CNS trajectories. However, key uncertainties remain, particularly regarding the causal role of senescence in human neurodegeneration, the specificity of current biomarkers, and the distinction between adaptive versus maladaptive senescence responses. Notably, direct evidence linking senescent cells to functional alterations in the human brain microenvironment remains limited. This review distinguishes itself from prior literature by integrating a multi-scale brain–body axis perspective, combining molecular, cellular, and systemic evidence to propose senescence as a bidirectional and context-dependent driver of neurodegeneration rather than a purely cell-autonomous process.

## Introduction

Aging is the strongest risk factor for neurodegenerative diseases such as Alzheimer’s Disease (AD), Parkinson’s Disease (PD), and Amyotrophic Lateral Sclerosis (ALS), which together represent a growing global health burden. The increasing life expectancy worldwide has led to a sharp rise in the prevalence of these disorders, with AD alone affecting over 55 million people globally and projected to exceed 139 million cases by 2050 (Hebert et al., 2013; Azam et al., 2021). While genetic mutations and protein aggregation have long been considered central to neurodegenerative pathology, emerging evidence highlights aging-related cellular processes as key drivers of disease progression (Shafqat et al., 2023; Baker and Petersen, 2018). Among the hallmarks of aging, cellular senescence has gained recognition as a major contributor to neurodegenerative decline, particularly through its role in neuroinflammation, mitochondrial dysfunction, and impaired proteostasis (Gillispie et al., 2021; Gaikwad et al., 2024).

Despite growing interest in cellular senescence in neurodegeneration, current frameworks remain fragmented, often focusing on isolated cellular compartments or single molecular pathways without integrating systemic contributions. In particular, the interaction between central and peripheral senescence, and its implications for disease propagation, remains poorly defined (Shafqat et al., 2023; Baker and Petersen, 2018).

This review addresses this gap by proposing a unified framework that integrates brain-intrinsic senescence with systemic aging processes across the brain–body axis, while critically examining unresolved mechanistic questions and translational limitations.

### Definition and identification of cellular senescence

Cellular senescence is classically defined as a stable and essentially irreversible cell cycle arrest triggered by a variety of stressors, including DNA damage, oxidative stress, telomere shortening, and oncogene activation (Campisi and d'Adda di Fagagna, 2007; He and Sharpless, 2017). However, the modern definition of senescence has evolved considerably, acknowledging its complexity, context-dependence, and the existence of heterogeneous phenotypes across tissues and cell types (Gorgoulis et al., 2019; Hernandez-Segura et al., 2018). Senescent cells often, but not always, express elevated levels of cyclin-dependent kinase inhibitors such as p16^INK4a, p21^Cip1/Waf1, or p19^Arf, exhibit DNA damage markers (e.g., γH2AX), senescence-associated *β*-galactosidase activity (SA-β-Gal), and develop a senescence-associated secretory phenotype (SASP) that includes pro-inflammatory cytokines, proteases, and growth factors (Campisi and d'Adda di Fagagna, 2007; Coppé et al., 2010; Kirkland and Tchkonia, 2020).

Despite these hallmark features, there is no single universal marker that defines senescence, and a combinatorial approach is generally required (Suryadevara et al., 2024; Suryadevara et al., 2024). In response to the lack of consensus, the Cellular Senescence Network (SenNet) Consortium recently proposed minimal criteria for the identification of senescent cells in human tissues and model organisms, emphasizing the need for context-specific marker panels and functional assays (Di Micco et al., 2021). These include not only molecular markers but also spatial and functional profiling, as senescence can occur in both dividing and non-dividing cells and exhibit tissue-specific phenotypes (Di Micco et al., 2021; Musi et al., 2018).

In the brain, this complexity is further amplified. Senescent-like phenotypes have been observed in glial cells (astrocytes, OPCS cells, microglia) and, more controversially, in post-mitotic neurons, where classical cell cycle arrest does not apply (Musi et al., 2018; Riessland et al., 2019). This calls for caution in extrapolating criteria from peripheral tissues to the CNS. Therefore, operational definitions of senescence in the brain must consider cellular identity, local microenvironment, and functional consequences, particularly when evaluating therapeutic interventions or biomarker development (Gorgoulis et al., 2019; Di Micco et al., 2021; Bussian et al., 2018).

### Neuronal senescence and the emergence of “neurescence”

Traditionally, cellular senescence has been considered a phenomenon exclusive to proliferative cells. Given that mature neurons are post-mitotic, their ability to undergo senescence was historically questioned. However, emerging evidence suggests that neurons can acquire a senescence-like phenotype in response to various stressors, a process now termed neurescence (Chou et al., 2023). This phenotype is characterized by the activation of senescence markers such as p21^Cip1, p16^INK4a, DNA damage foci, chromatin remodeling, and altered metabolic and secretory profiles, despite the absence of cell división (Musi et al., 2018; Riessland et al., 2019; Dehkordi et al., 2021). Such features have been identified in human and murine models of AD and PD, reinforcing the concept that neuronal senescence contributes to brain aging and pathology (Riessland et al., 2019; Bussian et al., 2018). Moreover, the SenNet Consortium recently issued guidelines formally recognizing neuronal senescence as a valid phenotype within the spectrum of cellular senescence in aging tissues (Suryadevara et al., 2024).

However, the classification of neurons as truly senescent remains debated. Unlike proliferative cells, neurons do not undergo classical cell cycle arrest, raising questions about whether these phenotypes represent bona fide senescence or senescence-like stress responses. Key distinguishing features between neurescence and chronic neuronal stress include the persistence and irreversibility of the phenotype, the presence of a functional SASP-like secretory profile, and the ability to influence the surrounding microenvironment. In contrast, transient DNA damage responses or metabolic dysfunction may mimic senescence markers without fulfilling these criterio (Riessland et al., 2019; Dehkordi et al., 2021).

Therefore, current evidence should be interpreted cautiously, and the term “senescence-like phenotype” may be more appropriate in certain contexts until standardized definitions are established.

### Glial senescence

Among the various cell types in the CNS, glial cells, particularly astrocytes, microglia, and oligodendrocyte precursor cells (OPCs), have been most consistently shown to undergo senescence with aging and in neurodegenerative conditions. Unlike neurons, which are post-mitotic and less prone to canonical forms of senescence, glial cells retain proliferative capacity and respond to diverse stressors by entering a senescent state characterized by metabolic changes, cell cycle arrest, and a pro-inflammatory secretory profile (Bussian et al., 2018; Hu et al., 2021; Sams, 2021).

Senescent astrocytes have been identified in both aged human and rodent brains, particularly in regions vulnerable to neurodegeneration such as the hippocampus and frontal cortex (Musi et al., 2018; Cohen and Torres, 2019). These astrocytes exhibit increased expression of senescence markers such as p16^INK4a, SA-*β*-gal activity, and *γ*-H2AX foci, and they secrete SASP components including IL-6, IL-1β, and matrix metalloproteinases (Bussian et al., 2018; Alshaebi et al., 2025). The accumulation of senescent astrocytes has been linked to impaired neurotrophic support, blood–brain barrier dysfunction, and synaptic dysregulation, contributing to neuronal vulnerability and disease progression (Alshaebi et al., 2025; Han et al., 2020). A key conceptual distinction must be made between glial activation and senescence. While activation is typically transient and reversible, senescence is characterized by persistent DDR signaling, stable phenotypic changes, and resistance to apoptosis. Chronic or unresolved activation may transition into senescence under sustained stress conditions; however, the molecular thresholds and temporal dynamics governing this transition remain poorly defined (Hernandez-Segura et al., 2018; Hu et al., 2021).

Microglia, the resident immune cells of the CNS, also develop a senescent-like phenotype with age. This phenotype, often referred to as “dystrophic” microglia, is characterized by cytoplasmic fragmentation, impaired phagocytic function, and heightened basal inflammatory activity (Mosher and Wyss-Coray, 2014; Mirarchi et al., 2024). Senescent microglia release SASP factors that sustain a chronic inflammatory milieu, exacerbating neuroinflammation and reducing the clearance of toxic aggregates such as Aβ and *α*-synuclein (Mirarchi et al., 2024; Martínez-Cué and Rueda, 2020). The identification of p16^INK4a-expressing microglia in aged murine models further supports their senescent status (Hu et al., 2021).

OPCs, a glial subpopulation responsible for myelin regeneration, have recently emerged as another target of senescence in aging and disease. OPCs exhibit increased DNA damage, reduced proliferation, and altered differentiation capacity with age, impairing remyelination and contributing to white matter pathology (Hudson et al., 2025; Zou et al., 2023; Neumann et al., 2019). Studies have shown that senescent OPCs secrete a distinct SASP, which includes factors that may inhibit myelin repair and further propagate local senescence through paracrine signaling (Zou et al., 2023).

Importantly, senescent glia are not isolated in their effects. The interplay between different senescent glial subtypes and their neighboring cells creates a feed-forward loop of inflammation and degeneration. For example, senescent astrocytes can prime microglia toward a pro-inflammatory phenotype, while SASP factors from microglia can reinforce astrocyte senescence and impair neuronal plasticity (Clarke et al., 2018; Boisvert et al., 2018; Hong et al., 2024). This reciprocal reinforcement amplifies the burden of senescence across the aging brain.

Altogether, the accumulation of senescent glial cells contributes significantly to the chronic inflammatory landscape of the aging CNS and plays a central role in driving neurodegenerative pathology. Targeting glial senescence, therefore, represents a promising therapeutic strategy to attenuate age-related CNS dysfunction and disease progression (Lau et al., 2023).

### Molecular features and functional consequences of senescent glial cells in the aging brain

Senescent glial cells in the aging brain exhibit a complex phenotype characterized by stable cell cycle arrest, resistance to apoptosis, metabolic reprogramming, and a persistent pro-inflammatory secretory profile collectively known as the previous mentioned SASP. This phenotype is orchestrated by key regulators such as p16^INK4a, p21^Cip1, and p53, which are activated in response to diverse cellular stressors including DNA damage, oxidative stress, and chronic inflammation (Hudson et al., 2025; Herranz and Gil, 2018; Liu, 2022). The accumulation of DNA damage foci and dysfunctional telomeres further sustains the senescent state through a chronic DDR, mediated by the ATM/ATR signaling pathways (Hudson et al., 2025; Liu, 2022; Ajoolabady et al., 2025).

Mechanistically, glial senescence can be conceptualized as a hierarchical cascade. Initial cellular stressors (e.g., oxidative stress, protein aggregation) trigger DNA damage responses (DDR), primarily via ATM/ATR signaling. Persistent DDR activation stabilizes cell cycle inhibitors such as p16 and p21, enforcing the senescent state. Subsequently, activation of transcriptional regulators such as NF-κB and C/EBPβ drives the establishment of the SASP, which in turn amplifies local inflammation and induces secondary senescence in neighboring cells. This feed-forward loop ultimately contributes to synaptic dysfunction, impaired proteostasis, and neuronal vulnerability (Coppé et al., 2010; Hudson et al., 2025; Liu, 2022).

The SASP secretome, which includes interleukins (e.g., IL-6, IL-1β), chemokines (e.g., CCL2/MCP-1, CXCL-1/3 and CXCL-10), growth factors (e.g., VEGF, GM-CSF), bioactive lipids, extracellular vesicles and matrix metalloproteinases, disrupts tissue homeostasis and may promote paracrine senescence in neighboring cells, although this mechanism remains largely supported by preclinical evidence and has not been definitively demonstrated in the human brain (Coppé et al., 2010; Lau et al., 2023; Yue et al., 2022). Notably, senescent astrocytes and microglia lose their neuroprotective roles, contributing to impaired synaptic plasticity, increased oxidative stress, and blood–brain barrier dysfunction (Hu et al., 2021; Alshaebi et al., 2025). OPC senescence further impairs remyelination and exacerbates white matter vulnerability, which is increasingly recognized as a core substrate of age-related cognitive decline and neurodegeneration (Arai, 2020).

**Figure 1:**
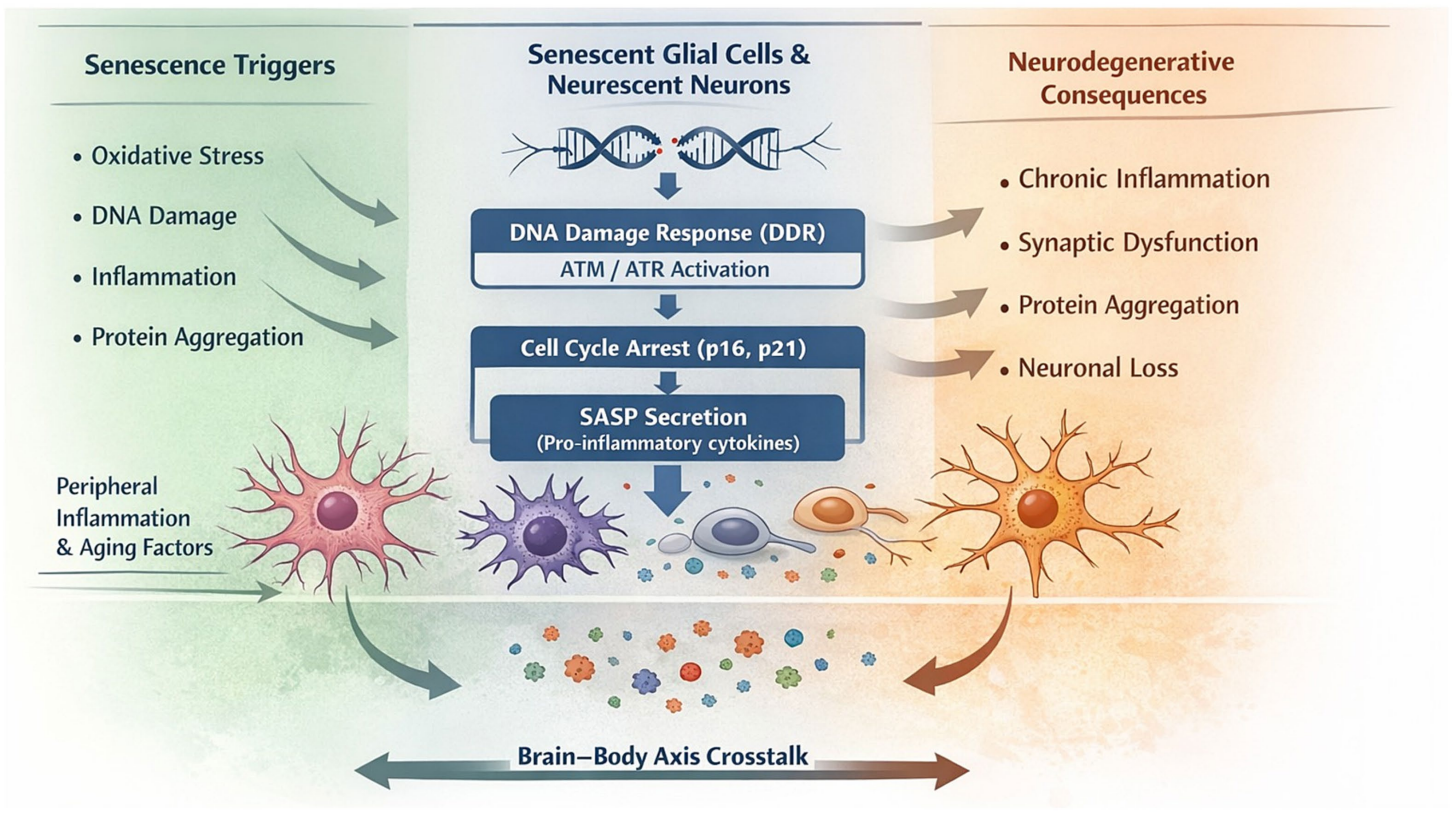
Senescence pathways in brain aging and neurodegeneration.

Although senescence was traditionally viewed as a protective response against tumorigenesis, its chronic persistence in non-regenerative tissues like the brain is now understood to drive maladaptive responses. In particular, senescent glial cells contribute to a chronic inflammatory milieu, termed “inflammaging,” which sensitizes the aging brain to neurodegenerative insults (Franceschi et al., 2018). Moreover, the SASP may synergize with disease-specific proteinopathies (e.g., tau, *α*-synuclein) by disrupting proteostasis and enhancing aggregation-prone environments, although direct causality remains to be fully elucidated (Musi et al., 2018; Bussian et al., 2018) (Figure 1).

### Neurescence: a distinct and emerging phenotype

Contrary to early assumptions that neurons, being terminally differentiated and post-mitotic, were immune to senescence, accumulating evidence has challenged this view. A growing body of work has identified features of cellular senescence in neurons, particularly in the aging brain and in neurodegenerative diseases such as AD and PD (Musi et al., 2018; Riessland et al., 2019; Dehkordi et al., 2021; Chinta et al., 2015).

Importantly, neurescent neurons display increased susceptibility to pathological protein aggregation, such as tau and *α*-synuclein, potentially linking senescence-like phenotypes to hallmark features of neurodegeneration (Riessland et al., 2019; Bussian et al., 2018; Dehkordi et al., 2021). These phenotypes may arise from persistent oxidative stress, telomere-associated DNA damage foci, and loss of proteostasis, rather than from replication-induced telomere shortening (Riessland et al., 2019; Bussian et al., 2018; Dehkordi et al., 2021; Jurk et al., 2012).

In AD, neurescence has been detected in hippocampal and cortical neurons expressing p16^INK4a and DNA damage markers such as γH2AX (Dehkordi et al., 2021). In PD, dopaminergic neurons of the substantia nigra exhibit senescence-associated chromatin alterations and upregulation of p21^Cip1, correlating with loss of function and neurodegeneration (Chinta et al., 2015; Rademacher et al., 2025). These findings suggest that neuronal senescence may contribute actively to disease progression, rather than being a mere consequence of age-related damage.

Despite this emerging evidence, the mechanistic drivers and functional consequences of neurescence remain under research. Whether these neurons are capable of triggering immune clearance, reprogramming, or exerting protective roles, as has been proposed for senescence in development and tissue remodeling, is still unclear. Further studies using single-cell profiling and *in vivo* lineage tracing will be crucial to delineate the biological relevance of neurescence in aging and disease.

### Targeting the SASP: pathways, senomorphic strategies, and therapeutic implications

Beyond their role in cell cycle arrest, senescent glial cells exert profound paracrine and systemic effects via the SASP. This secretory program is driven by sustained activation of several key pathways, including the NF-κB, p38 MAPK, mTOR, and cGAS-STING axes, which integrate stress signals and amplify pro-inflammatory gene expression profiles (Faget et al., 2019; Saito et al., 2024). These signaling cascades lead to the secretion of a complex mixture of cytokines, chemokines, growth factors, and matrix-remodeling enzymes, which reshape the brain microenvironment and facilitate inflammaging, synaptic dysfunction, and neuronal vulnerability (Faget et al., 2019; Saito et al., 2024; Alqahtani et al., 2025).

Unlike senolytic agents, senomorphic therapies aim to suppress or modulate the deleterious effects of the SASP without inducing cell death. This approach holds particular promise for the brain, where the irreversible loss of glial support functions could have detrimental effects. For example, rapamycin, an mTOR inhibitor, has been shown to reduce SASP output in astrocytes and microglia, alleviating neuroinflammation in models of aging and neurodegeneration (Selvarani et al., 2021; Johnson et al., 2015; Bielas et al., 2018). Similarly, JAK inhibitors such as ruxolitinib and p38 MAPK inhibitors have demonstrated efficacy in downregulating the SASP and restoring tissue homeostasis (Riessland et al., 2024; Riessland et al., 2024; Xu et al., 2015; Hongo et al., 2017).

Recent advances in transcriptomic and proteomic profiling have allowed the dissection of SASP components specific to different glial subtypes and disease stages. This opens the possibility for tailored interventions, targeting context-dependent SASP factors such as IL-6, MMP-3, or CXCL10, which are upregulated in AD and PD brains (Escartin et al., 2021; Evans et al., 2024).

Importantly, suppressing the SASP may also mitigate the propagation of secondary senescence, a phenomenon where SASP factors induce senescence in otherwise healthy neighboring cells. This feed-forward loop is particularly harmful in the CNS, where limited regenerative capacity amplifies the long-term consequences of glial senescence (Coppé et al., 2010). Therefore, senomorphic therapies represent a strategic avenue for halting the cascade of neuroinflammatory damage without compromising cellular integrity.

While clinical translation remains limited, the development of brain-penetrant senomorphics and biomarkers to monitor SASP suppression is an active area of research. Ultimately, integrating senomorphic strategies with disease-modifying therapies or regenerative approaches may offer synergistic benefits in slowing or reversing age-related cognitive decline (Saliev and Singh, 2025; Ji et al., 2023).

### Senolytic strategies in neurodegeneration: mechanisms, evidence, and translational challenges

Senolytics are pharmacological agents designed to selectively induce apoptosis in senescent cells by targeting their pro-survival pathways, collectively referred to as senescent cell anti-apoptotic pathways (SCAPs). In glial cells, SCAPs involve signaling via BCL-2 family proteins, PI3K/AKT, p53/p21, and HSP90, which are upregulated to counteract intrinsic apoptosis triggers (Zhu et al., 2015; Childs et al., 2017). Disruption of these pathways sensitizes senescent cells to cell death while sparing most healthy cells.

Preclinical studies have demonstrated the potential of senolytics in models of brain aging and neurodegeneration. For instance, the combination of dasatinib (a Src/tyrosine kinase inhibitor) and quercetin (a flavonoid with PI3K/AKT inhibitory activity) reduced astrocytic and microglial senescence in tauopathy mouse models, thereby improving synaptic density and cognitive performance (Riessland et al., 2019; Bussian et al., 2018; Riessland et al., 2024; Millar et al., 2025). Navitoclax (ABT-263), a BCL-2/BCL-xL inhibitor, has also shown an important role in neurovascular protection with significant cognitive benefit, but also the capacity to clear senescent OPCS and partially restore remyelination in aged rodents (Zhu et al., 2015; Tarantini et al., 2021; Zhu et al., 2016; Su et al., 2023).

Emerging senolytics with improved CNS penetration include fisetin, which has been reported to reduce neuroinflammation and improve behavior in aged mice, and FOXO4-DRI peptides, which disrupt the interaction between FOXO4 and p53, selectively inducing apoptosis in senescent cells (Zhu et al., 2015; Baar et al., 2017; Zhu et al., 2024). However, BBB penetration remains a major pharmacokinetic hurdle for many of these agents, and systemic administration carries the risk of off-target cytotoxicity.

In the context of AD, senolytic interventions have been shown to reduce tau pathology and attenuate the SASP, suggesting a synergistic relationship between senescent cell clearance and the mitigation of protein aggregation (Musi et al., 2018; Bussian et al., 2018; Zhu et al., 2024). In PD models, removal of senescent astrocytes improved dopaminergic neuron survival, highlighting disease-specific benefits of senolysis (Chinta et al., 2015; Miller et al., 2022).

Despite this promise, translation to human CNS diseases is in its infancy. The primary challenges include:

1. Achieving efficient and selective targeting of senescent cells within the brain without harming quiescent or transiently arrested cells.
2. Minimizing systemic toxicity, particularly thrombocytopenia observed with BCL-2 inhibitors.
3. Establishing reliable biomarkers to monitor treatment efficacy and target engagement *in vivo*.

Future strategies may involve localized delivery systems, nanocarrier formulations, or gene therapy-based approaches to enhance specificity. Furthermore, integrating senolytics with senomorphic agents or regenerative therapies may maximize benefits while minimizing risks.

### Biomarkers and monitoring strategies for cellular senescence in the human brain

The clinical translation of senescence-targeting therapies hinges on the development of reliable biomarkers capable of detecting, quantifying, and monitoring senescent cells *in vivo*. Unlike in peripheral tissues, where biopsies enable direct histological and molecular characterization, assessing cellular senescence in the CNS is constrained by its inaccessibility and cellular complexity.

Importantly, the identification of cell-type-specific senescence markers in the brain remains a major unmet need. Most currently used biomarkers lack specificity and may reflect overlapping biological processes such as inflammation, activation, or cellular stress (Suryadevara et al., 2024; Suryadevara et al., 2024; McCullumsmith et al., 2014; Baker et al., 2016).

Tissue-based biomarkers derived from postmortem or surgical samples remain the gold standard for validating senescence in the human brain. These include the expression of cyclin-dependent kinase inhibitors p16^INK4a and p21^Cip1, accumulation of DNA damage markers such as *γ*-H2AX and 53BP1 foci, and increased activity of senescence-associated *β*-galactosidase (SA-β-Gal) at suboptimal pH (Hernandez-Segura et al., 2018; McCullumsmith et al., 2014; Baker et al., 2016). However, their applicability in living patients is limited.

Fluid biomarkers offer a less invasive alternative. CSF analysis can detect SASP components, including IL-6, MCP-1/CCL2, and matrix metalloproteinases, which have been associated with both neuroinflammation and cognitive decline (Gaikwad et al., 2024; Barro et al., 2020; Dhauria et al., 2024). Plasma measurements of these factors, alongside extracellular vesicle cargo enriched in senescence-related microRNAs, have shown promise for reflecting CNS senescence status, although peripheral confounders remain a challenge (Guo et al., 2024).

On the other hand, neuroimaging approaches are an emerging frontier, and advanced MRI techniques, such as diffusion tensor imaging (DTI) and magnetic resonance spectroscopy (MRS), may indirectly capture structural and metabolic correlates of senescence-related pathology, including white matter deterioration and altered bioenergetics (Risacher and Saykin, 2013; Veeraiah and Jansen, 2023). Positron emission tomography (PET) tracers targeting glial activation such as TSPO ligands, are widely used as markers of glial-related signal; however, recent evidence suggests that TSPO-PET may reflect glial density or mitochondrial content rather than activation per se. Therefore, its interpretation as a marker of neuroinflammation or senescence should be approached with caution (Werry et al., 2019; Pan et al., 2024; Dupont et al., 2017; Nutma et al., 2021).

The integration of multi-omics profiling with longitudinal cognitive assessment represents a promising strategy for validating and tracking senescence biomarkers in at-risk populations. Combining transcriptomic, proteomic, and metabolomic data from blood and CSF may allow for the identification of robust biosignatures predictive of both disease progression and therapeutic response (Chen et al., 2023).

Ultimately, the establishment of validated biomarker panels will be essential in revealing insights for personalized health management (Babu and Snyder, 2023):

1. Identifying individuals who may benefit from senescence-targeting interventions.
2. Monitoring treatment efficacy and off-target effects.
3. Stratifying patients for clinical trials, thereby increasing the likelihood of detecting therapeutic benefit.

### Integrating biomarkers with interventions: clinical translation and actionable stratification

Taken together, fluid, imaging, and multi-omics biomarkers provide complementary, multi-scale readouts of senescence biology in the human brain. Fluid markers (SASP cytokines, EV-miRNAs, NfL, pTau217) capture systemic and CNS-derived signals; TSPO-PET and emerging SA-*β*-gal tracers index glial activation and senescence-like activity *in vivo*; and multi-omics frameworks delineate senescence-linked networks and molecular biotypes that map onto clinical phenotypes and risk trajectories (Barro et al., 2020; Dhauria et al., 2024; Guo et al., 2024; Werry et al., 2019; Pan et al., 2024; Dupont et al., 2017). Critically, this integrated biomarker toolkit can enable precision enrollment, target engagement, and response monitoring for senescence-targeting interventions; senolytics, senomorphics, and adjunctive new systemic approaches such as therapeutic plasma exchange with albumin replacement; thereby increasing the probability of success in early-stage, mechanism-anchored clinical trials (Selvarani et al., 2021; Johnson et al., 2015; Bielas et al., 2018; Riessland et al., 2024; Evans et al., 2024; Babu and Snyder, 2023; Saxton and Sabatini, 2017; Boada et al., 2019). In parallel, longitudinal designs that align biomarker dynamics with cognitive and imaging outcomes will be essential to determine when (preclinical vs. prodromal stages) and in whom senescence-modifying strategies yield the greatest benefit (Musi et al., 2018; Chou et al., 2023; Saliev and Singh, 2025).

An immediate translational goal is to develop composable panels that combine a small number of robust measures across modalities; for example, a plasma/CSF SASP index (IL-6, MCP-1, MMP-3/10), a TSPO-PET or alternative glial activation signal, and an omics-derived senescence metagene score; to stratify individuals by senescence burden, guide therapeutic pairing (senolytic vs. senomorphic ± disease-specific agents), and track on-target effects. Embedding these panels into proof-of-concept trials (including senolytics with CNS penetration, JAK/mTOR/p38 senomorphics, and TPE-albumin as a systemic anti-inflammatory/redox modulator) will operationalize a treat-to-biomarker paradigm for neurodegeneration (Selvarani et al., 2021; Johnson et al., 2015; Bielas et al., 2018; Riessland et al., 2024; Saliev and Singh, 2025; Boada et al., 2019).

### Integrating senescence-targeting therapies in neurodegeneration

The translation of senescence biology into clinical neurology requires not only the identification of therapeutic targets but also a framework for their integration into current treatment paradigms for neurodegenerative diseases. One of the central challenges is the timing of intervention, as senolytics and senomorphics are likely to be most effective at preclinical or prodromal stages, when senescent cell accumulation and SASP-driven inflammation are present but before irreversible neurodegeneration has occurred (Musi et al., 2018; Chou et al., 2023; Saliev and Singh, 2025). This highlights the importance of linking biomarker discovery with preventive strategies.

Another important perspective involves combinatorial approaches. Instead of seeing senescence clearance as a standalone strategy, future therapies might combine senolytics with:

- Regenerative interventions, such as stem cell transplantation and neurogenesis-promoting compounds, which could repopulate the niches left by senescent cell clearance (Chinta et al., 2015).
- Disease-specific treatments, including anti-amyloid or anti-tau therapies in AD and *α*-synuclein-targeting approaches in PD, where senescence may synergize with proteinopathy-driven pathology (Dehkordi et al., 2021).
- Metabolic modulators, such as mTOR inhibitors or NAD + boosters, which act systemically to delay senescence onset and enhance resilience to age-related stressors (Saxton and Sabatini, 2017). In this context, therapeutic plasma exchange with albumin replacement (TPE-albumin) represents a promising adjunctive intervention. Clinical evidence demonstrated that TPE with albumin not only reduced circulating toxic proteins and improved cognitive trajectories in AD, but also exerted systemic anti-inflammatory and redox-modulating effects (Boada et al., 2019). Such systemic interventions could synergize with senolytics or senomorphics by alleviating the pro-senescent milieu and enhancing overall brain resilience.

### Biomarkers of cellular senescence in brain aging and neurodegeneration

Identifying reliable biomarkers of cellular senescence in the central nervous system CNS is essential for translating basic mechanisms into clinical applications. Unlike peripheral tissues, where senescence markers can be directly interrogated in biopsies, the brain requires indirect yet robust approaches, including fluid biomarkers, molecular signatures, and neuroimaging readouts.

### Fluid biomarkers

CSF and blood represent the most accessible compartments to detect molecular correlates of senescence. Increased expression of p16^INK4a and p21^Cip1 transcripts in peripheral immune cells has been proposed as a systemic marker of organismal aging and neurodegenerative risk (Liu et al., 2009; Casella et al., 2019). In AD, CSF levels of neurofilament light (NfL) and pTau217 correlate with both neuronal damage and senescence-associated pathways, including DNA damage response and SASP-related cytokines (Mattsson et al., 2019; Palmqvist et al., 2020). Similarly, plasma IL-6, TNF-*α*, and MCP-1 are consistently elevated in patients with AD and PD, reflecting the pro-inflammatory milieu of senescent glial cells (Hu et al., 2019). Importantly, extracellular vesicles enriched in senescence-associated miRNAs have emerged as promising blood-based biomarkers, capable of reflecting ongoing senescence in astrocytes and microglia (Cheng et al., 2020).

### Imaging biomarkers

Molecular imaging provides unique opportunities to visualize senescence-related processes *in vivo*. TSPO-PET ligands, which are commonly interpreted as markers of microglial activation, although emerging evidence suggests they may more accurately reflect glial density or mitochondrial function (Kreisl et al., 2013; Corsi et al., 2022). Although not exclusive to senescence, TSPO upregulation overlaps with SASP-driven neuroinflammation. More recently, PET tracers targeting senescence-associated *β*-galactosidase activity have shown preclinical feasibility in identifying senescent cells *in vivo*, although their specificity is limited by the high baseline lysosomal β-galactosidase activity present in certain glial populations (Valieva et al., 2022). In addition, advanced MRI techniques detecting white matter microstructural damage and blood–brain barrier dysfunction provide indirect evidence of senescence-induced glial pathology, particularly in the context of aging and vascular contributions to cognitive decline (Montagne et al., 2015; Wharton et al., 2015).

### Omics approaches

High-dimensional omics technologies allow the characterization of senescence signatures at the molecular level. Transcriptomic and epigenomic profiling of aged human brains has revealed enrichment of senescence-related gene sets, including those linked to DDR, SASP, and mitochondrial dysfunction (Musi et al., 2018; Dehkordi et al., 2021). Proteomic studies have identified SASP, such as MMPs, complement factors, and pro-inflammatory cytokines, in both AD and PD brains, supporting their role as candidate biomarkers of cellular senescence and neurodegeneration (Panizza and Cerione, 2024). Recent analyses emphasize MMP-2, MMP-3, MMP-9, and particularly MMP-10 as early and progressive biomarkers associated with amyloid and tau pathology, BBB disruption, and neuroinflammation in AD, highlighting the clinical potential of integrating proteomic data for disease stratification (Radosinska and Radosinska, 2025). Metabolomic profiling further highlights perturbations in NAD + metabolism and redox homeostasis, consistent with senescence-driven metabolic reprogramming. Integrative multi-omic approaches combining genomics, proteomics, and metabolomics are beginning to define molecular biotypes of brain aging that may stratify individuals according to senescence burden and disease risk (Zhang et al., 2020; Franzmeier et al., 2020).

## Conclusions and future directions

The cumulative evidence reviewed herein consolidates cellular senescence as a key mechanistic bridge between aging and neurodegeneration, yet also reveals a landscape far more complex than initially anticipated. Far from being confined to neural tissues, senescence appears as a multi-compartmental and dynamic process, simultaneously shaped by local glial–neuronal crosstalk and by systemic factors originating in the periphery. While mounting experimental and translational data place senescence at the core of neurodegenerative cascades (Musi et al., 2018; Bussian et al., 2018; Dehkordi et al., 2021; Alshaebi et al., 2025), recent multi-cohort proteomic analyses now challenge the notion of a purely “central” mechanism.

In particular, large-scale plasma proteomic studies (Afshar et al., 2025) have shown that many peripheral proteins associated with AD endophenotypes are only weakly correlated with their brain counterparts, suggesting that peripheral proteostasis, metabolism, and immune activity may actively influence or even precede CNS senescence. This echoes previous parabiosis and therapeutic plasma exchange studies, where modifying the systemic milieu yielded measurable cognitive and inflammatory benefits without directly targeting the brain (Boada et al., 2019). Conversely, cutting-edge single-cell and spatial transcriptomic profiling (Wang et al., 2025) demonstrate that senescence phenotypes in neurons and glia are heterogeneous, region-specific, and not always mirrored by peripheral biomarkers, raising critical questions about causality, directionality, and compartmental independence.

Therefore, the present review contributes a synthesized framework that positions brain senescence not as an isolated pathology but as part of a systemic aging network, integrating genomic instability, immune signaling, and metabolic reprogramming across the brain–body axis. This systems-level perspective helps reconcile discrepancies between molecular, imaging, and clinical data, undersocring the necessity of multi-omic, longitudinal, and cross-compartment approaches to disentangle the interplay between central and peripheral senescence.

From a translational standpoint, senescence-targeting strategies (senolytics, senomorphics, metabolic modulators, or plasma-renewal interventions) should be designed and evaluated within this bidirectional framework. Future trials must determine whether reducing systemic senescence burden or reprogramming SASP signaling in the periphery can modify CNS trajectories, and whether central senescence can be accurately monitored through validated composite biomarkers integrating fluid, imaging, and omic readouts (Babu and Snyder, 2023; Panizza and Cerione, 2024; Radosinska and Radosinska, 2025; Zhang et al., 2020; Franzmeier et al., 2020).

Recent advances in the field have further refined our understanding of cellular senescence in the human brain. High-resolution single-cell and spatial transcriptomic studies have revealed a previously underappreciated heterogeneity of senescent-like phenotypes across neuronal and glial populations, challenging the notion of a uniform senescence program. In parallel, large-scale proteomic and multi-omic analyses have highlighted a partial dissociation between central and peripheral senescence signatures, reinforcing the concept of a bidirectional and compartmentalized brain–body axis. Moreover, emerging neuroimaging evidence suggests that commonly used proxies of neuroinflammation, such as TSPO-PET, may reflect glial density or mitochondrial function rather than activation per se, underscoring the need for more specific *in vivo* biomarkers of senescence. Together, these findings emphasize that while the field is rapidly evolving, fundamental questions regarding causality, specificity, and translational applicability remain unresolved (Suryadevara et al., 2024; Hudson et al., 2025; Riessland et al., 2024; Nutma et al., 2021; Wang et al., 2025).

Ultimately, the key frontier lies not in confirming that senescence is involved, but in clarifying where, when, and how it drives neurodegeneration, and whether modulating its systemic signatures can yield durable cognitive benefit. Bridging these questions through rigorous, multi-scale investigations will transform senescence biology from a conceptual paradigm into a clinically actionable framework for brain health and longevity.
